# Antineoplastic properties of polyphenols in TPC-1 human papillary thyroid carcinoma cell line: a systematic review

**DOI:** 10.20945/2359-3997000000645

**Published:** 2023-06-19

**Authors:** Marielle Lang Makiyama, Maria Júlia Pigatti Degli Esposti, Maria Luíza Raitz Siqueira, Margarete Dulce Bagatini, Sarah Franco Vieira de Oliveira Maciel, Marcelo Moreno

**Affiliations:** 1 Universidade Federal da Fronteira Sul Ciências Biomédicas Chapecó SC Brasil Ciências Biomédicas, Universidade Federal da Fronteira Sul, Chapecó, SC, Brasil; 2 Universidade Federal da Fronteira Sul Faculdade de Medicina Chapecó SC Brasil Faculdade de Medicina, Universidade Federal da Fronteira Sul, Chapecó, SC, Brasil; 3 Universidade Federal da Fronteira Sul Programa de Pós-graduação em Ciências Biomédicas Chapecó SC Brasil Programa de Pós-graduação em Ciências Biomédicas, Universidade Federal da Fronteira Sul, Chapecó, SC, Brasil

**Keywords:** TPC-1, polyphenols, thyroid cancer, papillary thyroid cancer

## Abstract

Thyroid cancer usually responds to surgical and ablative therapy, but when it's refractory the alternative lies in tyrosine kinase inhibitors that, in addition to harmful side effects, acts only in a palliative way. The concern for other therapeutic possibilities brought evidence on flavonoids, hypothesizing a possible strategy. This review aimed to organize a compilation of *in vitro* studies using polyphenol substances in TPC-1 (human papillary thyroid carcinoma cell line) summarizing it's results and describing the metabolic pathways involved. Articles were selected on PubMed, Google Scholar, LILACS, BVS and SciELO, using keywords “thyroid cancer”, “flavonoids” and “TPC-1”, until June 2022. 185 studies were selected. After identification and exclusion of duplicates and exclusion criteria applied, 11 original articles were evaluated. Of these, the findings of flavonoids added to TPC-1 were: inhibition of cell growth and viability, promotion of cell cycle arrest and induction of apoptosis. Polyphenolic compounds have antineoplastic properties by different mechanisms as shown *in vitro*, but the concentrations needed are above usual dietary consumption and the findings are limited to experimental cellular studies. Despite that, these results should be useful to guide further analysis aiming to reveal the real safety and efficacy of polyphenols in this scenario.

## INTRODUCTION

Thyroid cancer has had an increasing incidence in recent years, possibly due to the facility in performing cervical ultrasound examinations. However, mortality from this cause remained stable, since most thyroid neoplasms are differentiated carcinomas with an indolent behavior (1). The treatment of this condition consists mostly of surgical excision and, in some cases, complementation with radioactive iodine ablation. When patients have no response to this therapeutic regimen, the treatment possibilities are extremely limited, currently consisting in the use of tyrosine kinase inhibitors. This class of drugs, in addition to the harmful side effects worsening the quality of life of its users, acts in a palliative way, only delaying neoplastic proliferation and increasing survival (2,3). The search for other treatment possibilities in this limited scenario brought evidence on the use of polyphenol phytochemicals with antioxidant and antineoplastic actions, hypothesizing a possible therapeutic route. Cellular mechanisms, such as modifications in cell cycle, have already been described in several neoplastic cell lines using flavonoids (4), but in the field of thyroid cancer this evidence is still very scarce, consisting in studies using different flavonoid substances and different cell lineages. The Two-Pore Channel-1 (TPC-1) cell line was chosen in this review because it is one of the most prevalent cell lines in thyroid cancer, in which both *RET/PTC1* (rearranged during transfection/papillary thyroid carcinoma type 1) rearrangement and *BRAF V600E* (B-Raf proto-oncogene, serine/threonine kinase) mutations are present (most common genetic alterations detected in this kind of neoplasm) (4). The objective of this review was to organize a systematic review of data already evidenced by *in vitro* studies using TPC-1 cell line, providing a summary of the main results obtained and describing the metabolic pathways encountered, to enable future *in vivo* studies and clinical trials for contributing to develop new therapeutic strategies for patients which are refractory to conventional treatment.

## MATERIALS AND METHODS

A systematic review of the literature was carried out, designed, structured and registered based on the items of the Preferred Reporting Items for Systematic Reviews and Meta-Analyses (PRISMA) tool, and registered in the International Prospective Register of Systematic Reviews (PROSPERO) database (CRD42022340699).

### Search strategy and selection criteria

The search was applied to the MEDLINE databases via PubMed, Scientific Electronic Library Online (SciELO), Latin American and Caribbean Health Sciences Literature (LILACS), Virtual Health Library (BVS) and Google Scholar. The descriptors were “thyroid cancer” or “thyroid cancer – papillary”, “TPC-1” and “flavonoids” or “polyphenols”. All original articles that evaluated influences exerted by flavonoids on the TPC-1 cell line using *in vitro* models, published in Portuguese, English or Spanish, were included in the study.

No time limit was used for the publications included, as it is a recent topic in literature. Review articles, clinical studies, studies that did not evaluate the effect of flavonoids specifically in TPC-1 cell line, and, gray literature (letters to the editor, opinion articles, abstracts of scientific events), were excluded.

### Data collection and information quality assessment

After the initial search for descriptors in databases, the first analysis was performed to exclude duplicate studies. The remaining article contents were verified by title and abstract. This analysis determined the relevance of publications, and those that did not fit the selection criteria were also excluded.

The remaining articles were fully analyzed for the elaboration of this systematic review. After the complete reading of the texts, if any study still did not fit the selection criteria, it was also excluded, specifying the reason for the exclusion.

The manuscripts selected to compose this review had their data extracted into a spreadsheet (Microsoft® Excel version 16.42) based on the following domains: author names, year of publication, type of study, flavonoids used, concentrations of compounds, time of exposure to the compounds, cellular pathways evaluated and the effect of the polyphenols tested on cell lines.

The data obtained were presented through a table, as well as descriptive exposition and discussion of the results.

## RESULTS

**Figure 1:**
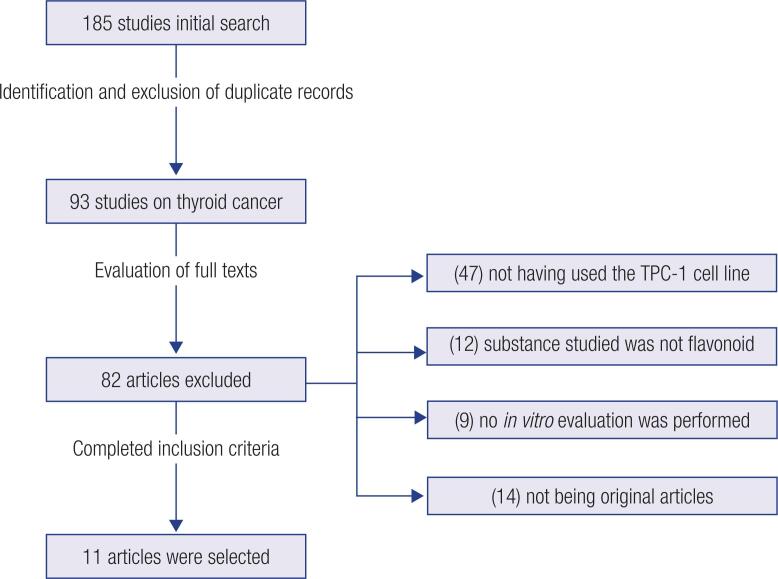
Flowchart for article selection.

The search resulted in 185 studies published from 1989 to 2022. After the identification and exclusion of duplicate records between the databases and the review studies, in addition to the exclusion of articles that did not use the thyroid cancer cell line criterion, 93 potential articles were evaluated by the titles and abstracts (Figure 1). After that, 82 articles were excluded by the following criteria: those that did not specifically use the TPC-1 cell line criterion (n = 47); the substance of interest was not a flavonoid (n = 12); no *in vitro* evaluation was performed (n = 9); articles that were not original (n = 14). Thus, the descriptive synthesis of this review consisted of 11 original articles, all with a pre-clinical design using the TPC-1 cell line criterion and different polyphenols (Figure 1).

The anticarcinogenic mechanisms of the flavonoid substances included in the studies selected were inhibition of cell proliferation, induction of apoptosis and cell cycle modifications, evidenced by methods such as: measurement of apoptotic proteins (caspases), cell cycle regulators (cyclin D1, c-Myc and β-catenin proteins, P*21 and P53* genes), activators or inhibitors of apoptosis (bax – bcl2 associated X, pro-caspase 3, bcl-2 – B-cell lymphoma 2, mcl-1 – induced myeloid leukemia cell differentiation, nuclear transcription factor – nrf2, survivin, poly(ADP-ribose) polymerase-PARP), inflammatory cytokines (tumor necrosis factor – TNFα), inductors of cell growth (vascular endothelial growth factor – VEGF and epidermal growth factor receptor – EGFR) and signaling pathways (mitogen activated protein kinase – MAPK/RAS-RAF-MEK extracellular signal-regulated kinases – ERK cascade and janus kinase – JAK/signal transducer and activator of transcription – STAT-3 pathway) (5–15).

**Figure 2:**
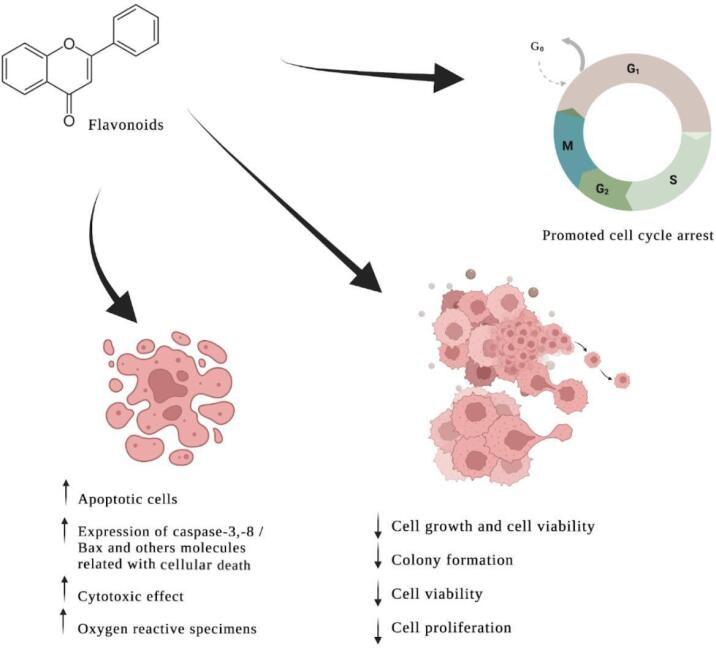
Summary of polyphenols mechanisms of influence on TPC-1 cell line.

The main results from the selected articles are demonstrated in Table 1 and Figure 2.

**Table 1:** Main results about the mechanisms underlying the TPC-1 cell line and the use of flavonoids

References	Flavonoid molecule(s) and concentrations	Analysis methods	Main results
Kang and cols., 2011	Genistein – 72 h exposition with 10-50-100 μM/mL	MTT assay	Inhibition of cell growth in a dose dependent mode
	Resveratrol – up to 120 h exposition with 50 μM/mL	MTT assay	Inhibition of cell growth
	Quercetin – 72 h exposition with 10-50-100 μM/mL	MTT assay	Inhibition of cell growth in a dose dependent mode
Bian and cols., 2020	Resveratrol – 48 h exposition with up to 50 μM/mL	MTT assay	Decrease of cell viability in a dose dependent mode
		Flow cytometry	Cell cycle arrest and increase of apoptotic cells
		Colony formation	Colony formation decrease
		Western blotting	Increase of caspase-3,-8 and bax expression, and decrease of bcl-xl and mcl-1 expression
Perna and cols., 2018	Curcumin – 48 h exposition with 25 μM/mL	Immunoblotting	Degradated nrf2 and reduced VEGF expression
		Immunoblotting	Up regulation of TNFα in 2 of the 3 complexes tested
		MTT assay	Reduced cell viability
		Western blotting	Down regulation of cell cycle regulators (cyclin D1, β-catenin, p21, p53) and activators or inhibitors of apoptosis (pro-caspase3, bcl-2)
Esposito and cols., 2019	Curcumin – 48 h exposition with 25 μM/mL	MTT assay	Cell viability reduction
		Immunofluorescence	Damage of the cell nucleus
		Western blotting	Down regulation of cell cycle regulators (cyclin D1, β-catenin, *P21, P53*) and activators or inhibitors of apoptosis (pro-caspase3, bcl-2)
Zhou and cols., 2019	Naringin – 6, 12 or 25 μM/mL, dose- and time-dependently	MTT assay	Cell proliferation inhibition
		Flow cytometry	Cell apoptosis induction
		Western blotting	Enhancement of Caspase3 and bax expression, and reduction of cyclin D1, c-Myc, survivin, and bcl-2 expression; suppression of PI3K/AKT pathway activation
Orlandella and cols., 2022	Extract from *Annurca Flesh Apple* (AFPE) – 24 h exposition with up to 250 μM/mL	Flow cytometry	Reduction of cell viability and promotion of cell cycle arrest
	Extract from AFPE – 20 h exposition with 500 μM/mL	DCFDA/H2DCFDA- cellular ROS assay kit	Reduction of cell death induced by oxidative stress
Wu and cols., 2019	Epigallocatechin-3-gallate (EGCG) – 24 h exposition with doses from 10-200 μM/mL	EdU and MTS assays	Inhibition of cell proliferation and cell viability in a dose dependent mode
		Flow cytometry	Cell cycle arrest
	Up to 25 μM/mL of EGCG	TUNEL staining and western blotting	Increase of the apoptotic index in a dose dependent mode
	Up to 50-200 μM/mL EGCG	Western blotting	Increase of the bax/bcl-2 ratio and the expression levels of cleaved caspase-3 and cleaved PARP
	50-200 μM/mL EGCG	Western blotting	Gradually decrease of p-EGFR, RAS, p-RAF, p-MEK1/2, and p-ERK1/2
Yin and cols., 2017	*Prunella vulgaris* (PV) – Rutin, hyperoside and other flavonoids isolated and identified from PV: luteolin, homoorinetin, cinaroside, quercetin and kaempferol. Concentrations of 0, 5, 10, 20 and 30 % for 12, 24 and 36 h	CCK-8 assay	Cell proliferation inhibition
	IC50 PV for 24 h	Hoechst 33342 staining	Alteration of cellular morphology and cell nuclei
		DNA extraction and fragmentation assay	Induction of cell apoptosis
		RT-qPCR	Decrease bcl-2 expression and increase bax and caspases-3 expression
Liang and cols., 2020	Fisetin from 1 μM/mL to 30 μM/mL for 24 h	MTT assay	Promotion of a cytotoxic effect in a dose dependent mode
	15 μM/mL for 24 h	Morphological examination in fluorescence microscope	Induction of cell apoptosis without compromising cellular membrane
	20 μM/mL for 24 h	Morphological examination in fluorescence microscope	Elevated quantity of late apoptotic cells - strong effect on the nucleus morphology related to apoptosis
		DCFH-DA dye	ROS enhancement
		Spectrofluorimetry	Reduction of the mitochondrial membrane potential
	15-20 μM/mL for 24 h	Flow cytometry	Alteration of cell cycle progression in a dose dependent mode
		Western blotting	Down regulation of the cyclin D1 expression; up regulation of STAT3 and decreased expression of JAK-1
		ELISA	Elevated protein expression of cleaved caspase-3, and −9 in a dose dependent mode
Oh and cols., 2013	Silibinin – 100 μM/mL for 24 h	Quick cell proliferation assay kit II	Decrease in cell viability
Carvalho and cols., 2018	Xanthohumol – 10-100 μM/mL for 48 h	Sulforhodamine B assay	Decrease in cell viability in a dose dependent mode
	100 μM/mL for 48 h	TUNEL assay	High cell death rate and high percentage of fragmented DNA
	Concentrations up to 10 μM/mL for 48 h	Western blotting	Increase in caspase-3 and caspase-7 expression
	10 μM/mL for 72 h	Flow cytometry	Cell cycle arrest
	0.05-0.1-100 μM/mL for 72 h	Fenton reaction initiated deoxyribose degradation cell-free assay	Radical levels increase at higher concentrations (pro-oxidant effect)

Acronyms: MTT (3-(4,5-dimethylthiazol-2-yl)-2,5-diphenyl-2H-tetrazolium bromide); bax (bcl2 associated X), bcl-xl (B-cell lymphoma); mcl-1 (induced myeloid leukemia cell differentiation); nrf2 (nuclear transcription factor −2); VEGF (vascular endothelial growth factor); TNFα (tumor necrosis factor α); ROS (Reactive Oxygen Species); PI3K/AKT (phosphatidylinositol-3-kinase/protein kinase B); AFPE (*Annurca Flesh Apple*); DCFDA/H2DCFDA – Cellular ROS (2’,7’-dicholofluorescin diacetate and reactive oxygen species); EGCG (Epigallocatechin-3-gallate); EdU; (5-ethynyl-2’-deoxyuridine); MTS [3-(4,5-dimethylthiazol-2-yl)-5-(3-carboxymethoxyphenyl)-2-(-4sulfophenyl)-2H-tetrazoluim, inner salt]; TUNEL (terminal deoxynucleotidyl transferase dUTP nick end labeling); PARP [poly(ADP-ribose) polymerase]; EGFR (epidermal growth factor receptor); RAS (rat sarcoma); RAF (rapidly accelerated fibrosarcoma); MEK (mitogen-activated protein kinase kinase); ERK (extracellular signal-regulated kinase); PV (*Prunella vulgaris*); CCK-8 assay (cell counting kit-8); DNA (deoxyribonucleic acid); STAT3 (signal transducer and activator of transcription 3): JAK-1 (janus kinase-1); RT-qPCR (Reverse transcription-quantitative polymerase chain reaction).

Created by the authors using Biorender.

## DISCUSSION

### Thyroid cancer

Thyroid cancer is considered the most common endocrine neoplasm in the world. According to the Global Cancer Observatory, its incidence in 2020 was 6.6 per million individuals, being the fifth most common malignancy diagnosed in women. Papillary thyroid carcinoma represents about 90% of all thyroid cancers (1). Studies report that this increasing incidence may be related to greater medical vigilance and improved diagnosis, mainly associated with the introduction of new technologies and more availability of ultrasound examinations. Besides that, other factors such as obesity, iodine intake, environmental exposure to chemicals, use of fertility drugs (such as clomiphene citrate and progesterone) and insulin resistance syndrome may also have potential contributions (16,17). According to Borges and cols., this neoplasm is predominant in fertile women.

Thyroid cancer is divided into subtypes according to the degrees of cell differentiation and their histological and pathological features, which have strong influence in disease progression and treatment strategies. Among the thyroid cancer subtypes, the well-differentiated carcinomas are the most frequent, such as papillary and follicular thyroid cancer. The other tumors correspond to medullary and anaplastic carcinomas that tend to be more aggressive and less responsive to treatment (18). The average age of diagnosis for well-differentiated carcinomas is between 45 and 55, and, among the main symptoms, thyroid cancer is usually characterized by the presence of nodules, swelling and asymmetry of the neck, but, in most cases, may be completely asymptomatic (19).

Several aspects can increase the risk of developing this pathology, including a history of radiation exposure, a family history of thyroid cancer in a first-degree relative and some genetic conditions, such as multiple endocrine neoplasia syndrome. The main treatment for thyroid cancer is surgery (total or partial thyroidectomy), and other postoperative interventions may be needed according to tumor characteristics, such as radioactive iodine therapy (3).

In 2017, the US Preventive Services Task Force published a review against screening thyroid nodules in an asymptomatic general adult population, for it ended up causing greater harm compared to the expected benefits, mainly due to unnecessary surgery and postoperative complications (2).

Papillary thyroid carcinoma (PTC) is the most common histological subtype, accounting for 90% of cases. It develops in follicular cells and has a benign behavior (20). Most cases involving this carcinoma present a positive evolution when treated correctly, with low mortality rates. However, in refractory cases, five-year survival is <50% and has an estimated incidence of 4-5 cases per million, resulting in impaired quality of life and possible unfavorable outcomes (21,22). These cases usually involve PTC variants, which have more aggressive clinicopathological features and are associated with higher mortality due to their high metastatic rate, such as the micropapillary variant (23), solid variant (24) and tall cell variant (25).

In recent years, protein tyrosine kinase inhibitors have emerged as an alternative for treatment in refractory cases of PTC. They target intracellular tyrosine kinase (TK), a protein that catalyzes protein substrates and modulates enzymatic activity, positively interfering in various mechanisms of tumor growth and spread, such as angiogenesis, lymphangiogenesis and proliferation of cancer cells. Different TK inhibitors, such as Subnitib®, Sorafenib® and Vandetanib®, act on several TKs, inhibiting target cells in a non-selective manner. For this reason, severe side effects may occur due to toxicity associated with the difficulty in selecting specific targets and to the use of high doses needed because of the possibility of developing drug resistance (26,27).

### Flavonoids

Flavonoid is the nomenclature used to define a class of polyphenolic substances of plant origin (28). The basic chemical structure of this group, which is the most representative class of phyto phenolic compounds, is composed of two aromatic rings linked by three carbons, which can convert to an oxygenated heterocyclic structure (C6-C3-C6). They are found in a wide variety of foods, such as fruits, vegetables, wine, chocolate and tea (Table 2) (29).

**Table 2:** Flavonoids with substance examples and their food sources

Subclass	Substances	Source
Flavanols	Epigallocatechin, catechin, epicatechin	Food (astringency, bitterness, sourness, viscosity, aroma and color features)
Flavonols	Quercetin, kaempferol, myricetin, rutin	Cherries, grapes, apricots, red wine, chocolate and tea
Anthocyanidins	Pelargonidin, cyanidin, malvidin	Vegetables (colors blue, purple and red)
Flavones	Luteolin, apigenin, chrysin	Fruit and vegetables
Isoflavones	Genistein, daidzein	Leguminous plants
Flavonones	Naringenin, hesperidin, eriodictyol	Citrus

Adapted from: Cosme and cols., 2020 (31); De Souza Dos Santos and cols., 2011 (32).

In this context, there are divergences in the literature below regarding the dosage of these compounds in daily diet, since this value is influenced by factors such as: age and sex of the population, method of analysis for absorption in each individual and even annual season and soil characteristics, since they cause differences in the quality of flavonoid substances. Some studies have shown considerable differences in the amount of flavonoid intake per day, which values can vary from 23.0 mg to 1.0 g or 1.1 g (30,31).

It is also worth highlighting the classification of these substances, since structural differences at the molecular level can promote different biological effects (32). Regarding the therapeutic potential of this polyphenolic class, studies indicate its importance in several scenarios, both for treatment and prevention of diseases. In that context, its potential effect on cardiovascular diseases, cancer, gastric and duodenal ulcers, vascular fragilities and as antibacterial and anti-allergic treatments were described (33).

Among the biological effects mentioned – one of great value, especially regarding cancer therapy – is the antineoplastic potential of flavonoids due to their antiproliferative activity. In this sense, many studies reveal the inhibition of cell transformation and proliferation with the administration of flavonoid substances. A North American study describes the influence of some different classes of polyphenols as inhibitors of neoplastic transformation, highlighting the diverse action mechanisms of these compounds used individually or combined (34). Furthermore, a European study shows that flavonoids have the potential to inhibit cell growth, especially in the context of colorectal cancer. Thus, while inhibiting cell proliferation of the epithelium and inducing the process of apoptosis, such substances may have therapeutic potential (35).

### Therapeutic potentials

Regarding flavonoids and the concentrations used in the studies, genistein and quercetin were administered by Kang and cols. (2011) in a 72-hour exposition with concentrations of 10, 50 and 100 μM/mL. Both compounds promoted inhibition of cell growth in a dose-dependent mode in the MTT (3-(4,5-dimethylthiazol-2-yl)-2,5-diphenyl-2H-tetrazolium bromide) assay. In the same study, exposition of resveratrol in a concentration of 50 μM/mL for up to 120 hours showed an inhibition of cell growth (5).

Resveratrol was also used by Bian and cols. (2020) at a concentration of up to 50 μM/mL in a 48 hours exposition, and presented different results according to the method of analysis applied. The analysis from the MTT assay revealed a decrease in cell viability in a dose dependent mode; in the flow cytometry method, cell cycle arrest and increase of apoptotic cells was observed; in the Colony formation, a colony formation decrease was found. Western blotting analysis showed an increase of caspase-3, −8 and bax expression, and a decrease of bcl-xl and mcl-1 expression (7).

Perna and cols. (2018) evaluated curcumin with a concentration of 25 μM/mL in a 48-hour exposition, showing different cellular responses according to the method of analysis. In the Immunoblotting method, three results were obtained: degraded nrf2, reduced VEGF expression and up-regulated TNFα in 2 of the 3 complexes tested. In the analysis from the MTT assay, there was a reduced cell viability, and in the Western blotting analysis, both cell cycle regulators (cyclin D1, β-catenin, *P21, P53*) and apoptosis regulators (pro-caspase3, bcl-2) were down-regulated (10).

Curcumin was also used by Esposito and cols. (2019) in a 48-hour exposition with a concentration of 25 μM/mL. The MTT assay revealed cell viability reduction, and immunofluorescence analysis demonstrated damage to the cell nucleus. There was a down regulation of cell cycle regulators (cyclin D1, β-catenin, p21, p53) and apoptosis regulators (pro-caspase3, bcl-2), verified by Western blotting, similar to that observed by Perna and cols. (2018) (9,10).

Zhou and cols. (2019) used narigin at concentrations of 6, 12 or 25 μM/mL. In the MTT assay, cell proliferation inhibition was observed, and flow cytometry showed an induction in cell apoptosis. In the Western blotting analysis, there was an enhancement of caspase 3 and bax expression, reduction of cyclin D1, c-Myc, survivin, and bcl-2 expression, as well as the suppression of PI3K/AKT pathway activation (12).

Using extract from Annurca Flesh Apple (AFPE), Orlandella and cols. (2022) applied it in a 24 hours exposition with a concentration of up to 250 μM/mL, resulting in a reduction of cell viability and promotion of cell cycle arrest analyzed by flow cytometry. Another concentration of AFPE (500 μM/mL) in a 20 hours exposition was evaluated by the DCFDA/H2DCFDA – Cellular ROS Assay Kit, obtaining a reduction of cell death induced by oxidative stress, contrasting with the findings described so far (13).

Epigallocatechin-3-gallate (EGCG) showed different cellular responses when used in different concentrations in the study by Wu and cols. (2019). Considering 24 hours of exposure with doses between 10-200 μM/mL, it inhibits cell proliferation and cell viability when analyzed by EdU and MTS assays methods, and causes cell cycle arrest as shown in flow cytometry. In the same article, concentration up to 25 μM/mL, evaluated by TUNEL staining and Western blotting, revealed an increase of the apoptotic index. Concentrations up to 50-200 μM/mL, using Western blotting method, showed an increase of the bax/bcl-2 ratio and the expression levels of cleaved caspase-3 and cleaved PARP. Furthermore, Western blotting was used to analyze exposure with 50-200 μM/mL EGCG, which revealed a gradually decrease of p-EGFR, RAS, p-RAF, p-MEK1/2 and p-ERK1/2 (11).

In the study by Yin and cols. (2017) *Prunella vulgaris* (PV) was used, a compound with rutin, hyperoside and other flavonoids isolated and identified from PV (luteolin, homoorinetin, cinaroside, quercetin and kaempferol). At concentrations of 0, 5, 10, 20 and 30% for 12, 24 and 36 hours, based on a CCK-8 assay, cell proliferation inhibition was observed. When used 50% PV for 24 hours, the Hoechst 33342 staining method revealed alteration of cellular morphology and cell nuclei, DNA extraction and fragmentation assay showed induction of cell apoptosis and RT-qPCR indicated decrease of bcl-2 expression and increase of bax and caspase-3 expression (8).

Fisetin was used in the research conducted by Liang and cols. (2020) at concentrations from 1 μM/mL to 30 μM/mL for 24 hours, promoting a cytotoxic effect evidenced by the MTT assay. At 15 μM/mL dose, through the morphological examination in a fluorescence microscope, induction of cell apoptosis without compromising cellular membrane was visualized. In the concentration of 20 μM/mL, still using the same analysis, an elevated quantity of late apoptotic cells was observed. The same dose revealed ROS enhancement, shown by DCFH-DA dye method, and reduction of the mitochondrial membrane potential was detected by spectrofluorimetry. The concentration of 15-20 μM/mL was analyzed by flow cytometry which showed an alteration of cell cycle progression in a dose dependent mode. The Western blotting method revealed a down regulation of the cyclin D1 expression; up regulation of STAT3 and decreased expression of JAK-1. ELISA analysis showed an elevated protein expression of cleaved caspase-3, and −9 in a dose dependent mode (6).

Silibinin was applied by Oh and cols. (2013) with a 100 μM/mL dose for 24 hours, which revealed a decrease in cell viability with the quick cell proliferation assay kit II (14). Xanthohumol was used in the research by Carvalho and cols. (2018) for two 48-hour and 72-hour time slots of exposure. For the first time slot, 10-100 μM/mL concentration showed, in sulforhodamine B assay, a decrease in cell viability in a dose dependent mode. Concentrations of 100 μM/mL revealed a high cell death rate and high percentage of fragmented DNA in the TUNEL assay, and finally in doses of up to 10 μM/mL demonstrated an increase in caspase-3 and caspase-7 expressions using the western blotting method. For the second time slot another 10 μM/mL concentration was applied, which revealed a cell cycle arrest using flow cytometry. Finally, exposure to doses of 0.05-0.1-100 μM/mL showed increased levels of ROS at higher concentrations (pro-oxidant effect) through a fenton reaction and deoxyribose degradation cell-free assay (15).

In summary, all polyphenol substances studied had a molecular influence in the TPC-1 cell lineage, mostly in a dose and time dependent manner. Cellular proliferation was inhibited in all studies analyzed, most of them showed by the MTT assay (5-7,9,10,12,14,36). Cell cycle arrest was another mechanism evaluated primarily by using flow cytometry (37). The majority of polyphenols increased the activity of caspases (6–12,15) and down-regulated the activity of cyclin D1, leading to cell death (6,9,10,12). The induction of apoptosis, evaluated by flow cytometry (7,12), TUNEL (terminal deoxynucleotidyl transferase dUTP nick end labeling) assay (11), DNA extraction and fragmentation assay (8), and analysis through morphological examination in a fluorescence microscope (6) found DNA damage (8,15) and accumulation of ROS (6,15). In contrast, only one study using pretreatment with AFPE showed a reduction in cell death induced by oxidative stress, underlying that polyphenols found specifically in this source may (alone or in combination) show a protective effect in this specific scenario (13). Finally, cell cycle and apoptosis regulators, inflammatory cytokines, inductors of cell growth, signaling pathways and altered mitochondrial membrane potentiality were also associated to the causing of cell death in TPC-1 lineage (6–13,15).

Therefore, it is possible to consider that many polyphenolic compounds have antineoplastic properties specifically in TPC-1 cell lineage. *In vitro* studies obtained results by using concentrations above usual dietary consumption, and tested the toxicity in control cells, without evidence of cell damage. However, this result is limited to cellular analysis, and this evidence should be used for studies in animal models, aiming to know the real safety and efficacy of these substances in this scenario. According to this evidence, some studies have already tested these polyphenolic compounds associated with antineoplastic drugs and the results, so far, are promising. For example, cisplatin administered with curcumin in mice potentiates the cytotoxicity via mediating cell death and cycle arrest, enhancing drug sensitivity and preventing resistance, in addition to reduced cisplatin-mediated side effects (38). Specifically, on thyroid cancer, a study using PTC cell culture of thyroidectomized patients showed that the addition of the isoflavone daidzein conjugated with an anti-estrogenic compound (N-t-boc-hexylenediamine) was able to amplify the inhibitory effect of Sorafenib® on cells, with a dosage ten times lower, reducing its important side effects (39). Another study, using PTC cell lineages K1 and BCPAP, showed that the co-administration of polyphenol quercetin and Sorafenib® lead to a reduction of the proliferation, adhesion and migration properties of the cells, enabling the reduction of the effective anticancer dosage of the drug (40).

Although relevant, these findings are reflected in *in vitro* studies using high doses of phytochemicals. Their usual consumption was not able to demonstrate protection or risk of inducing neoplasms, whether thyroid or other primary foci. In addition to the amount of exposure to the phytochemical, one must consider the factors that influence the extraction of the active ingredient, which vary according to location, sun exposure, soil characteristics, among other factors. Therefore, to extrapolate these findings to all substances, it would be necessary to rigidly standardize the substances and adequately measure it in individuals who, in addition to the confounding factors related to the phytochemical, also suffer influences in the individual's organism, where absorption can be influenced by intestinal flora, interactions with other medications and other possible influencers. Therefore, an animal model with greater possibility of controlling individual factors and a standardization of the substance would be necessary to carry out more in-depth studies on this topic (41,42).

In conclusion, there is evidence that polyphenolic compounds have antineoplastic properties on a cellular level, through mechanisms such as inhibiting cell growth, increasing expression of apoptotic proteins, stimulating down regulation of cell cycle regulators and causing DNA damage, but, so far, in doses above those usually obtained through dietary consumption. There is also no evidence of toxicity considering cellular models. These findings reveal a possible therapeutic potential of polyphenols due to their direct effects on neoplastic cells and their actions combined with antineoplastic drugs. However, these results deserve further studies, advancing to trials in animal models and in association with other substances such as tyrosine kinase inhibitors, and, seeking alternatives for the treatment of refractory cases, which are extremely limited in current treatments.
